# Unveiling the hidden: a deep learning approach to unraveling subzone-specific changes in peripapillary atrophy in type 2 diabetes

**DOI:** 10.3389/fcell.2024.1459040

**Published:** 2024-08-27

**Authors:** Yingying Li, Xinxin Hu, Xinyu Guo, Xueqiong Ye, Dandan Wang, Juntao Zhang, Weina Ren, Na Zhao, Yitian Zhao, Qinkang Lu

**Affiliations:** ^1^ Department of Ophthalmology, The Affiliated People’s Hospital of Ningbo University, Ningbo University, Ningbo, China; ^2^ Ningbo Clinical Research Center for Ophthalmology, Ningbo, China; ^3^ Ningbo Key Laboratory for Neuroretinopathy Medical Research, Ningbo, China; ^4^ Eye Hospital of Wenzhou Medical University (Ningbo Branch), Ningbo, China; ^5^ Laboratory of Advanced Theranostic Materials and Technology, Ningbo Institute of Materials Technology and Engineering, Chinese Academy of Sciences, Ningbo, China

**Keywords:** deep learning, peripapillary atrophy, diabetic retinopathy, gamma zone, beta zone

## Abstract

**Purpose:**

This study aimed to evaluate the optical coherence tomography angiography (OCTA) changes in subzones of peripapillary atrophy (PPA) among type 2 diabetic patients (T2DM) with or without diabetic retinopathy (DR) using well-designed deep learning models.

**Methods:**

A multi-task joint deep-learning model was trained and validated on 2,820 images to automate the determination and quantification of the microstructure and corresponding microcirculation of beta zone and gamma zone PPA. This model was then applied in the cross-sectional study encompassing 44 eyes affected by non-proliferative diabetic retinopathy (NPDR) and 46 eyes without DR (NDR). OCTA was utilized to image the peripapillary area in four layers: superficial capillary plexus (SCP), deep capillary plexus (DCP), choroidal capillary (CC) and middle-to-large choroidal vessel (MLCV).

**Results:**

The patients in both groups were matched for age, sex, BMI, and axial length. The width and area of the gamma zone were significantly smaller in NPDR group compared to the NDR group. Multiple linear regression analysis revealed a negative association between the diagnosis of DR and the width and area of the gamma zone. The gamma zone exhibited higher SCP, DCP and MLCV density than the beta zone, while the beta zone showed higher CC density than the gamma zone. In comparison to the NDR group, the MLCV density of gamma zone was significantly lower in NPDR group, and this density was positively correlated with the width and area of the gamma zone.

**Discussion:**

DR-induced peripapillary vascular changes primarily occur in gamma zone PPA. After eliminating the influence of axial length, our study demonstrated a negative correlation between DR and the gamma zone PPA. Longitudinal studies are required to further elucidate the role of the gamma zone in the development and progression of DR.

## 1 Introduction

The global incidence of diabetes has been increasing in recent years. According to the Diabetes Atlas 10th edition, the prevalence of diabetes is expected to reach 783 million by 2045 (Sun et al., 2023). Diabetic retinopathy (DR) is one of the major microvascular complications in diabetic patients and has become a leading cause of irreversible visual impairment and blindness among the adult working population and elderly individuals worldwide (Yau et al., 2012). Recent studies have suggested that retinal nerve fiber layer and retinal ganglion cells are damaged at an early stage of DR, indicating that DR is a vascular neuropathy (Chhablani et al., 2015; Vujosevic et al., 2018).

Although several studies have demonstrated diabetic macular vascular changes (Sun et al., 2019; You et al., 2020), the evidence regarding the associations between peripapillary vascular changes and the pathogenesis of DR has been limited. Previous studies found a negative correlation between the presence of peripapillary atrophy (PPA)-β and the prevalence of DR, independent of axial length (AL) and refractive status (Tan et al., 2018). However, that study did not quantitatively determine the microstructure and microcirculation of PPA-β, which limited further exploration of the association between PPA-β and DR. Additionally, PPA-β has been found to be associated with the incidence and progression of glaucoma (Jonas and Naumann, 1989; Lee et al., 2011; Uchida et al., 1998). Considering that diabetes is a recognized high-risk factor for glaucoma, and altered ocular microcirculation is one of the common causes in both diseases, it is reasonable to speculate that PPA-β and corresponding vascular metrics are associated with DR.

PPA-β, characterized by visible large choroidal vessels and sclera, has recently been subdivided into a new beta zone and a gamma zone based on the Bruch’s membrane opening (BMO) (Jonas et al., 2012; Dai et al., 2013). The gamma zone represents the peripapillary area free of Bruch’s membrane (BM), while the new beta zone is defined as the continued presence of BM and absence of retinal pigment epithelium (RPE). Previous studies have reported the microvascular features of PPA subzones in myopic eyes using spectral domain optical coherence tomography angiography (SD-OCTA) (Hu et al., 2021). To our knowledge, there have been no studies exploring the changes in PPA subzones secondary to DR. In this study, we introduce a multi-task joint deep-learning framework based on state-of-the-art models to achieve automated determination and quantification of the microstructure and microcirculation of the beta zone and gamma zone PPA in diabetes with or without DR.

## 2 Methods

### 2.1 Study design and participants

In this cross-sectional study, we adhered to the principles outlined in the Declaration of Helsinki and obtained approval from Ethics Committee of the Ophthalmology Center of The Affiliated People’s Hospital of Ningbo University, China. Written informed consent was obtained from all participants.

The inclusion criteria for all subjects were as follows: 1) individuals with type 2 diabetes mellitus (T2DM) without diabetic retinopathy (NDR) or with untreated non-proliferative diabetic retinopathy (NPDR). The diagnosis of T2DM followed WHO standards (Alberti and Zimmet, 1998), while the grading of NPDR was based on the International Clinical DR Disease Severity Scale (Wilkinson et al., 2003). 2) Presence of PPA (beta zone, gamma zone, or both).

The exclusion criteria were as follows: 1) intraocular pressure (IOP) > 21 mmHg; 2) presence of other chorioretinal diseases, glaucoma, optic neuropathy, or systemic diseases that may affect ocular perfusion; 3) history of intraocular surgery including cataract surgery, retina laser and intravitreal injections. 4) presence of mental illness or inability to cooperate with the examination; 5) poor quality of OCTA images and structural OCT images. Once the binocular parameters met the above criteria, one eye per participant was randomly selected.

### 2.2 Data acquisition

A detailed questionnaire collected personal information including age, gender, medical history, and history of eye disease/surgery. General examinations measured height, weight, systolic blood pressure (SBP) and diastolic blood pressure (DBP) for each participant. DR was diagnosed and graded using digital fundus photography (Canon CR-2; Tokyo, Japan). The fundus camera captured two images, one centered on the macula and the other on the optic disc. Axial length and IOP were measured using the IOLMaster (Carl Zeiss Meditec AG, Jena, Germany), and the noncontact tonometer (Auto Tonometer TX-F; Topcon, Tokyo, Japan), respectively. To calculate the patients’ body mass index (BMI), their weight was divided by their height in meters squared (kg/m^2^). The mean arterial pressure (MAP) and ocular perfusion pressure (OPP) were calculated using the following formulas: MAP = 1/3SBP+2/3DBP and OPP = 2/3MAP-IOP (Longo et al., 2004).

### 2.3 OCT angiography image acquisition

For the acquisition of peripapillary vascular imaging, we utilized SS-OCT angiography (SS-OCTA) with the BM-400K BMizar from TowardPi Medical Technology Co., Ltd. in Beijing, China. The imaging camera employed a vertical cavity laser with a wavelength of 1,060 nm and a scanning speed of 400 KHz A-scan/s. This setup allowed for a lateral resolution of 10 μm and an axial resolution of 3.8 μm (optical), enabling the visualization of *en face* images of the retinal and choroidal vascular perfusion system. To evaluate the peripapillary area of each subject, B-scans were performed, covering a 6 mm^2^ × 6 mm^2^ area horizontally and vertically.

To stratify the retinal and choroidal vasculature, we utilized the built-in software in the OCTA system. This software allowed us to segment the vasculature into several sub-layers, including the superficial capillary plexus (SCP), deep capillary plexus (DCP), choroidal capillary (CC), and middle-to-large choroidal vessel (MLCV). In our study, we excluded images with significant motion artifacts or those graded below 8. Manual correction was only performed when automatic stratification was inaccurate.

All OCTA images were acquired by an experienced operator (YL) and reviewed by two other authors (XH and NZ).

### 2.4 Multi-task joint deep-learning framework

#### 2.4.1 PPA segmentation

**FIGURE 1:**
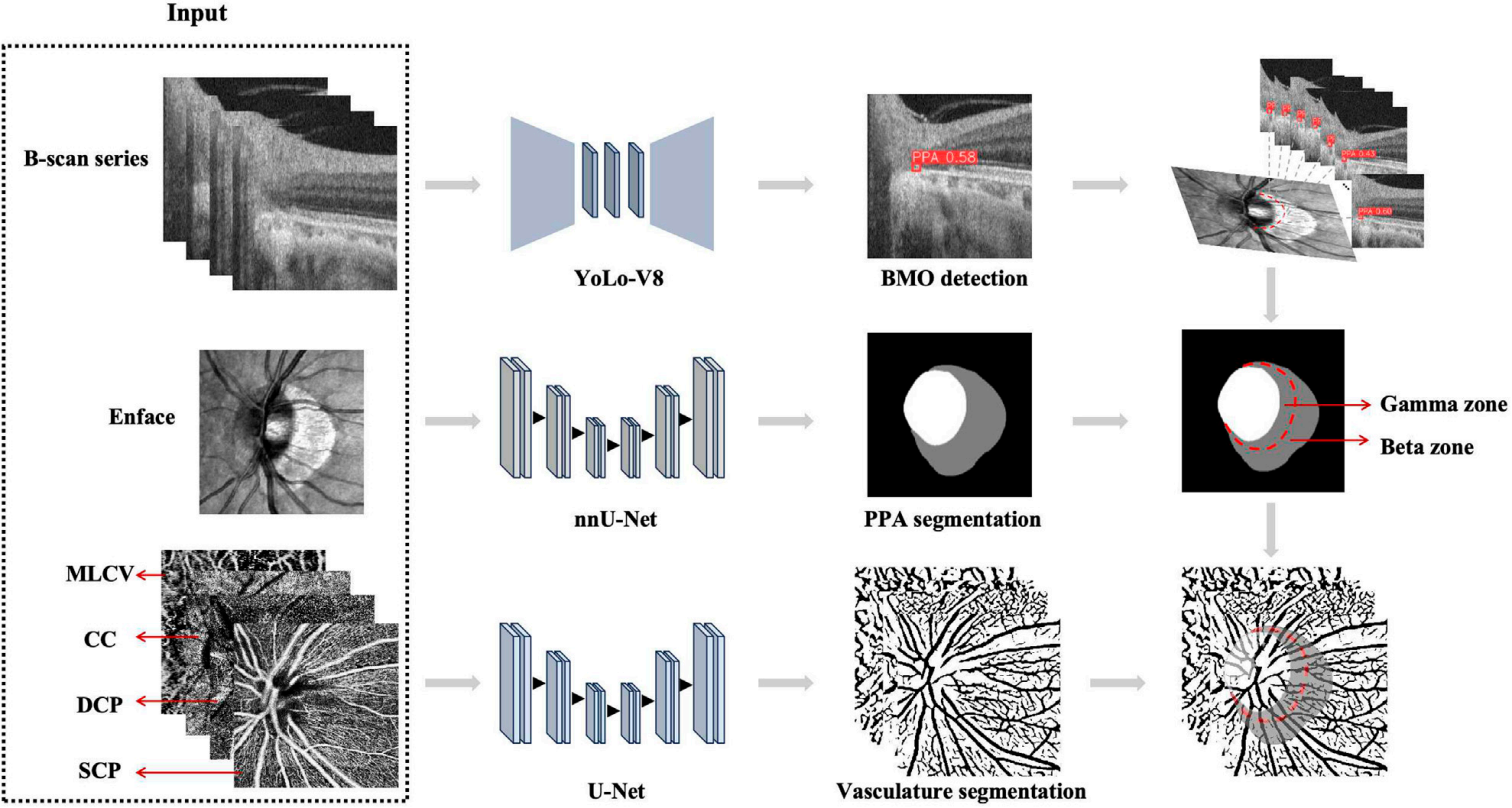
The workflow of the beta zone and gamma zone PPA automated detection. Firstly, the nnU-Net was used for segmenting the border of optic papilla and RPE atrophy. Then the YoLo-V8 was employed to detect the Bruch’s membrane opening (BMO) on B-scan series. Lastly, the BM boundary curve was obtained by reconstructing the BMO points from B-Scans onto enface images and fitting them with a polynomial function.

The beta zone PPA is defined as the presence of BM without the RPE. On the other hand, the gamma zone PPA is characterized by the absence of BM and is located between the optic papilla border and beta zone. To accurately delineate the boundaries of the optic disc, beta zone, and gamma zone PPA, both *en face* and serial B-scan OCT images of the optic disc were acquired at the same position, as shown in Figure 1. For the segmentation of the optic papilla border and RPE atrophy, we employed an advanced image segmentation algorithm called nnU-Net (Isensee et al., 2021). We chose the Stochastic Gradient Descent (SGD) with Nesterov momentum of 0.99 as optimizer and the initial learning rate was set to 0.001 in our experiment. The total epochs reached 1,000, with each epoch consisting of 250 iterations. To train and validate this model, we utilized an additional dataset of 200 *en face* images that were manually annotated. The training and validation sets were partitioned in a 7:3 ration, and the segmentation performance demonstrated its robustness and effectiveness, with a Dice coefficient greater than 0.95.

#### 2.4.2 BMO detection

To accurately detect the BMO on B-scan series, we utilized YoLo-V8 (Wang et al., 2024), an end-to-end object detection model that excels in real-time processing of high-resolution images. This model has been widely applied in the field of medical image detection, enabling precise target identification. Prior to implementation, YoLo-V8 was trained and validated on 20 subjects, consisting of 2,500 manually annotated B-scan images. We chose the Stochastic Gradient Descent (SGD) with Nesterov momentum of 0.937 as optimizer and the initial learning rate was set to 0.001. The maximum number of epochs reached 300. The BM boundary curve was obtained by reconstructing the BMO points from the B-Scans onto enface images and fitting them with a polynomial function. Subsequently, the width and area of PPA subzone were calculated using MATLAB software.

#### 2.4.3 Vessel segmentation and quantification

**FIGURE 2:**
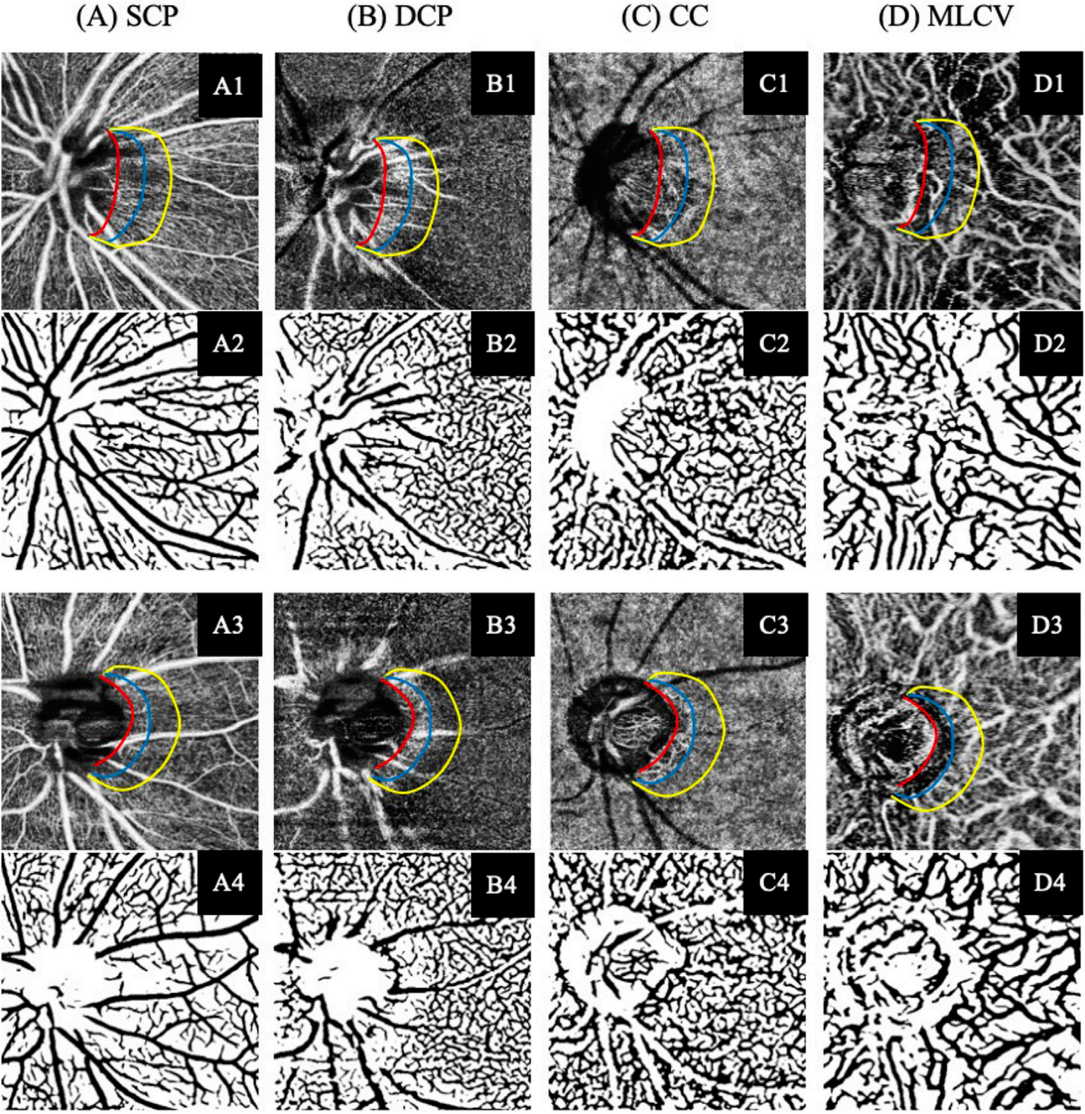
Illustration of the SS-OCTA imaging and vascular segmentation of peripapillary vasculature parameters in each retina and choroidal layer. **(A)** Superficial capillary plexus (SCP). **(B)** Deep capillary plexus (DCP). **(C)** Choroidal capillary (CC). **(D)** Middle-to-large choroidal vessel (MLCV). (A1–D1) SS-OCTA images of participant without DR. (A3–D3) SS-OCTA images of participant with NPDR. (A2–D2, A4–D4) The corresponding automated segmented vessel map of A1–D1 and A3–D3, respectively.

We utilized an enhanced U-Net model for the segmentation of vessel in the SCP, DCP, CC and MLCV of OCTA images (Figure 2). This model incorporated a U-Net (Ronneberger et al., 2015) as the underlying architecture, with the original encoder was replaced by a pre-trained ResNet18 (He et al., 2016). We chose the AdamW as optimizer and the initial learning rate was set to 0.0002. The maximum number of epochs reached 200. To train the network, we employed a proprietary OCTA vessel segmentation dataset called OCTA-Z. OCTA-Z comprising 126 OCTA *en face* angiograms from 42 subjects, with a resolution of 512 × 512 pixels. The vessel annotation at the pixel-level was performed by four proficient image experts, followed by a review and refinement of the initial labeling by two senior ophthalmologists. For this study, we allocated 120 OCTA images from 30 subjects for training the improved U-Net, while the remaining images were used for testing. The network achieved a Dice coefficient of 81.86% and an accuracy is 90.13% on the testing set. Vascular density (%) was determined as the proportion of the measured area occupied by flowing blood vessels within a specific region. We measured the vascular density of four retinal and choroidal sub-layers in beta zone and gamma zone PPA, respectively.

### 2.5 Statistical analysis

All statistical analyses were performed using SPSS version 29.0 (SPSS, Chicago, IL, United States). Continuous data were presented as mean ± standard deviation, while categorical data were reported as frequency and percentage (%). Parametric data were analyzed using Student’s t-test, while non-parametric data were analyzed using the Mann–Whitney *U* test was for group comparisons. The Chi-squared test was employed to compare categorical variables. The gamma zone indicators that exhibited significant differences between the two groups were selected as dependent variables, and stepwise linear regression analysis was conducted to identify independent risk factors. Additionally, partial correlation coefficient analyses were carried out to assess the effects of PPA subzone parameters on ocular parameters after adjusting for axial length. All P-values were two-sided, and statistical significance was determined when the P-values were <0.05.

## 3 Results

The study consisted of 46 eyes from 46 subjects without DR (NDR group) and 44 eyes from 44 subjects with NPDR (NPDR group). Among the participants, 54 (60.0%) were male and 36 (40.0%) were female. The mean age was 60.3 ± 9.8 years (median, 60 years; range, 33–85 years), and the mean axial length was 24.1 ± 1.1 mm (median, 23.9 mm; range, 22.3–27.4 mm). All subjects had a diagnosis of T2DM, and their eyes were phakic.

### 3.1 Comparison of demographic and clinical characteristics between the groups

Table 1 provides a summary of the comparison of demographic and clinical characteristics between the NDR group and NPDR group. The two groups were matched for age, sex, BMI and axial length. Significant differences were observed in fasting plasma glucose (FPG), glycated hemoglobin (HbA1c), and urine albumin-to-creatinine ratio (UACR) between the two groups.

**TABLE 1:** Demographic and clinical characteristics of the participants enrolled in this study.

Variables	NDR (n = 46)	NPDR (n = 44)	*P*-value
Age (years)	60.1 ± 10.4	60.5 ± 9.2	0.84^†^
Male (%)	22 (47.8)	32 (72.7)	0.25*
BMI (kg/m^2^)	24.3 ± 2.6	24.9 ± 3.2	0.37^†^
Axial length (mm)	24.2 ± 1.1	23.9 ± 1.0	0.16^‡^
IOP (mmHg)	15.2 ± 3.0	15.1 ± 2.6	0.82^‡^
OPP (mmHg)	50.6 ± 8.2	51.3 ± 8.0	0.78^†^
DBP (mmHg)	78.9 ± 9.6	80.8 ± 9.6	0.38^†^
SBP (mmHg)	131.8 ± 17.8	136.7 ± 18.0	0.20^†^
MAP (mmHg)	96.4 ± 11.5	99.4 ± 11.5	0.23^†^
FPG (mmol/L)	7.0 ± 2.3	8.1 ± 1.9	<0.001^‡^
HbA1c (%)	6.5 ± 1.4	7.5 ± 1.5	<0.001^‡^
UACR (mg/g)	14.0 ± 18.4	139.3 ± 275.3	<0.001^‡^
TC (mmol/L)	4.6 ± 1.0	4.9 ± 1.0	0.12^†^
TG (mmol/L)	1.8 ± 1.1	2.0 ± 2.2	0.94^‡^
HDL (mmol/L)	1.3 ± 0.2	1.3 ± 0.3	0.61^†^
LDL (mmol/L)	2.3 ± 0.7	2.6 ± 0.7	0.09^†^

BMI, body mass index; IOP, intraocular pressure; OPP, mean ocular perfusion pressure; DBP, diastolic blood pressure; SBP, systolic blood pressure; MAP, mean arterial pressure; FPG, fasting plasma glucose; HbA1c, glycosylated hemoglobin; UACR, urine albumin creatine ratio; TC, total cholesterol; TG, triglycerides; HDL, high-density lipoprotein; LDL, low-density lipoprotein.

* chi-square test.

^†^ Student’s t-test.

^‡^ Mann-Whitney *U* test.

The FPG of the NPDR group (8.1 ± 1.9 mmol/L; range, 4.9–14.6 mmol/L) was significantly higher (*P <* 0.001) than that of the NDR group (7.0 ± 2.3 mmol/L; range, 4.2–14.7 mmol/L). Similarly, mean HbA1c (7.5% ± 1.5%) and UACR (139.3 ± 275.3 mg/g) in the NPDR group were significantly higher (all *P* < 0.001) than those in the NDR group (6.5% ± 1.4% and 14.0 ± 18.4 mg/g). No significant differences were observed between the two groups in terms of IOP, OPP, DBP, SBP, MAP, total cholesterol (TC), triglycerides (TG), high-density lipoprotein (HDL), and low-density lipoprotein (LDL) levels (all *P* > 0.05).

### 3.2 Comparison of beta and gamma zone characteristics between the groups

In this study, a total of 60 eyes had a beta zone, with 30 (50.0%) eyes in the NDR group and 30 (50.0%) eyes in the NPDR group (*P* = 0.77). Additionally, 60 eyes had a gamma zone, with 32 (53.3%) eyes in the NDR group and 28 (46.7%) eyes in the NPDR group (*P* = 0.55) (Table 2). The mean width of the gamma zone was significantly smaller in the NPDR group (0.39 ± 0.14 mm) compared to the NDR group (0.52 ± 0.18 mm) (*P <* 0.001). Similar statistically significant differences were observed in the gamma zone area, where individuals in the NPDR group exhibited a smaller gamma zone area (0.57 ± 0.17 mm^2^) compared to those in the NDR group (0.96 ± 0.44 mm^2^) (*P <* 0.001). Furthermore, the vascular density of MLCV in the gamma zone was significantly lower in the NPDR group (22.90% ± 10.54%) compared to the NDR group (29.36% ± 8.11%) (*P* = 0.005).

**TABLE 2:** Comparison of beta and gamma zone PPA between the NDR and NPDR groups.

Variables	NDR (n = 46)	NPDR (n = 44)	*P*
Eyes with beta zone (%)	30 (65.2)	30 (68.2)	0.77*
Eyes with gamma zone (%)	32 (69.6)	28 (63.6)	0.55*
Width of beta zone (mm)	0.45 ± 0.10	0.44 ± 0.13	0.70^†^
Area of beta zone (mm^2^)	0.84 ± 0.26	0.80 ± 0.32	0.54^†^
Width of gamma zone (mm)	0.52 ± 0.18	0.39 ± 0.14	<0.001^†^
Area of gamma zone (mm^2^)	0.96 ± 0.44	0.57 ± 0.17	<0.001^‡^
Beta zone vascular density (%)			
Superficial capillary plexus (SCP)	31.64 ± 5.80	31.08 ± 5.38	0.34^‡^
Deep capillary plexus (DCP)	34.01 ± 6.09	33.42 ± 3.94	0.66^†^
Choroidal capillary (CC)	49.39 ± 6.42	49.91 ± 5.69	0.74^†^
Middle-to-large choroidal vessel (MLCV)	19.78 ± 7.99	20.90 ± 10.20	0.64^†^
Gamma zone vascular density (%)			
Superficial capillary plexus (SCP)	35.43 ± 5.78	32.99 ± 6.85	0.24^‡^
Deep capillary plexus (DCP)	39.41 ± 6.52	40.21 ± 5.81	0.31^†^
Choroidal capillary (CC)	39.94 ± 7.31	38.75 ± 9.08	0.29^†^
Middle-to-large choroidal vessel (MLCV)	29.36 ± 8.11	22.90 ± 10.54	0.005^†^

* chi-square test.

^†^ Student’s t-test.

^‡^ Mann-Whitney *U* test.

**FIGURE 3:**
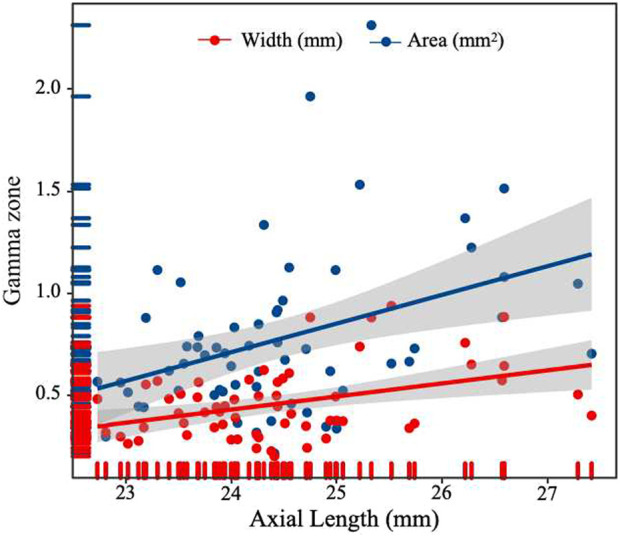
Scatterplot showing the correlation between width and area of gamma zone PPA and axial length.

After conducting a multiple forward stepwise linear regression analysis, we examined the relationship between the width and area of the gamma zone PPA and several independent variables, including age, sex, BMI, FPG, HbA1c, UACR, TC, diagnosis of DR, and axial length (Table 3). The findings revealed significant associations between these variables and the width and area of gamma zone. Specifically, the diagnosis of DR was found to have a negative association with both the width and area of the gamma zone (*P* = 0.005 and *P <* 0.001, respectively). This suggests that individuals with a diagnosis of DR tend to have narrower and smaller gamma zones. On the other hand, longer axial length was positively associated with the width and area of gamma zone (*P* = 0.005 and *P* = 0.007, respectively) (Figure 3). This indicates that individuals with a greater axial length tend to have wider and larger gamma zones. These findings provide valuable insights into the relationships between the diagnosis of DR, axial length, and the characteristics of the gamma zone.

**TABLE 3:** Stepwise linear regression analysis of predictors for width and area of gamma zone PPA in all subjects.

	*R* ^2^	Independent Factor	Unstandardized coefficients				
			*B*	SD	SC Beta	*t*	*P*
Gamma zone width	0.272	Axial length	0.054	0.018	0.339	2.953	0.005
		Diagnosis of DR	−0.117	0.040	−0.339	−2.954	0.005
Gamma zone area	0.338	Axial length	0.112	0.040	0.308	2.810	0.007
		Diagnosis of DR	−0.345	0.086	−0.441	−4.023	<0.001

SC, standardized coefficients.

### 3.3 Analyses of parapapillary vascular parameters in beta and gamma zone

Table 4 presents the parapapillary vascular differences between beta zone and gamma zone. The SCP density was 31.36% ± 5.55% in the beta zone and 34.29% ± 6.37% in the gamma zone, showing a statistically significant difference (*P* = 0.003). Similarly, the DCP density in the gamma zone (39.78% ± 6.16%) was significantly higher than that in the beta zone (33.72% ± 5.10%) (*P* < 0.001). The DCP density of the gamma zone PPA was negatively correlated with the width (*P* = 0.003) and area (*P <* 0.001) of the gamma zone (Table 5).

**TABLE 4:** Comparison of parapapillary vascular density in beta zone and gamma zone PPA.

Variables	Beta zone	Gamma zone	P-value
Corresponding subzone width (mm)	0.44 ± 0.12	0.46 ± 0.17	0.88^‡^
Corresponding subzone area (mm^2^)	0.82 ± 0.29	0.78 ± 0.39	0.09^‡^
Corresponding retinal vascular density (%)			
Superficial capillary plexus (SCP)	31.36 ± 5.55	34.29 ± 6.37	0.003^‡^
Deep capillary plexus (DCP)	33.72 ± 5.10	39.78 ± 6.16	<0.001^†^
Corresponding choroidal vascular density (%)			
Choroidal capillaries (CC)	49.65 ± 6.02	39.38 ± 8.14	<0.001^‡^
Middle-to-large choroidal vessel (MLCV)	20.34 ± 9.10	26.35 ± 9.80	<0.001^†^

^†^ Student’s t-test.

^‡^ Mann-Whitney U test.

**TABLE 5:** Correlation with the width and area of beta and gamma zone PPA.

Variables	Beta width		Beta area		Gamma width		Gamma area	
	*r*	*P* ^a^	*r*	*P* ^a^	*r*	*P* ^a^	*r*	*P* ^a^
Corresponding retinal vascular density (%)								
Superficial capillary plexus (SCP)	−0.06	0.67	−0.02	0.87	0.05	0.69	0.04	0.74
Deep capillary plexus (DCP)	−0.02	0.86	−0.16	0.22	−0.38	0.003	−0.42	<0.001
Corresponding choroidal vascular density (%)								
Choroidal capillaries (CC)	−0.47	<0.001	−0.45	<0.001	−0.04	0.78	−0.09	0.49
Middle-to-large choroidal vessel (MLCV)	0.14	0.29	0.17	0.20	0.37	0.004	0.48	<0.001

^a^ *P*-value adjusted for axial length.

Regarding the choroidal metrics, the beta zone exhibited a higher CC density (50.96% ± 6.93%) compared to the gamma zone (38.72% ± 8.68%), with a statistically significant difference (*P* < 0.001). The CC density in the beta zone PPA was negatively correlated with the width (*P* = 0.029) and area (*P <* 0.001*)* of the beta zone. However, the MLCV density in the gamma zone (26.35% ± 9.80%) was significantly higher than that in the beta zone (20.34% ± 9.10%) (*P* < 0.001). Notably, the MLCV density of gamma zone PPA was positively correlated with the width (*P =* 0.004) and the area (*P <* 0.001) of the gamma zone.

## 4 Discussion

This study represents the first investigation into the impact of DR on beta and gamma zone PPA. To achieve automated determination and quantification of the microstructure and microcirculation of the corresponding beta zone and gamma zone PPA, we employed a state-of-the-art deep learning approach. Our findings indicate that patients with DR had a significantly smaller gamma zone PPA compared to those without DR, but no significant difference was observed in the beta zone PPA. Additionally, we observed topographic differences in the SCP, DCP, CC and MLCV between the beta zone and gamma zone PPA. The gamma zone PPA exhibited higher densities of SCP, DCP and MLCV, while the beta zone displayed higher CC density compared to the gamma zone.

The gamma zone PPA is characterized by atrophy of the RPE and BM. Previous studies have suggested a close relationship between the formation and development of the gamma zone PPA and AL (Jonas et al., 2016; Zhang et al., 2018). Our study confirmed a similar result, showing that longer axial length was associated with the width and area of the gamma zone. Furthermore, our multiple forward stepwise linear regression analysis revealed that the presence of DR independently increased the risk factor of a narrower and smaller gamma zone PPA. This aligns with a study by Lin et al. (2022), who reported a significantly smaller PPA-β area in children with diabetes compared to healthy controls. However, their study did not differentiate between the beta zone and gamma zone PPA, limiting the further exploration. Considering that the choroidal circulation serves as the primary source of oxygen and nutrients for the choroid and outer layers of the retina, one may speculate that the effect of DR on the gamma zone PPA could be associated with hyperglycemia-induced choroidal alterations. Previous studies have indicated larger peripapillary choroidal thickness (pCT) in individuals with diabetes compared to healthy individuals (Li et al., 2020). It is possible that the increased supply of peripapillary blood flow during the early stage of DR inhibits atrophic changes to BM, thus slowing down the expansion of the gamma zone PPA.

The beta zone PPA is defined as absence of RPE with continued presence of BM. Previous studies have shown that beta zone PPA is associated with age and the presence of glaucoma (Dai et al., 2013; Kim et al., 2014). Sung et al. (2018) found that myopic eyes with PPA + BM (indicating beta zone PPA in this study) had the lowest superficial radial peripapillary capillary (RPC) and deep vessel density, which were closely negatively correlated with the width of the beta zone. Similarly, we previously reported that the beta zone PPA in myopic eyes had the lowest superficial RPC and choroidal microvasculature compared to the other subzones of PPA, and these parameters were negatively associated with the width and area of the beta zone PPA (Hu et al., 2021). This study is partially consistent with these findings, as we observed lower SCP, DCP and MLCV densities in the beta zone compared to the gamma zone. Both SCP and DCP densities were negatively associated with the width and area of the beta zone PPA, indicating a microcirculatory deficiency in the retinal layer of the beta zone PPA. However, the density of CC measured in our study was significantly lower than that reported in previous studies. This difference could be attributed to the fact that the participants in those studies were normal myopic eyes with a mean age of no more than 30 years. Furthermore, diabetes and its associated ocular complication, DR, are recognized risk factors for increased peripapillary CC flow deficit percentage (CC FD%) (Guo et al., 2023; Wang et al., 2023). It is important to note that the SS-OCTA we utilized in our study showed higher sensitivity and image resolution in distinguishing between CC and MLCV compared to conventional SD-OCTA, which may explain the differences in outcome.

The gamma zone PPA is primarily formed by oblique border tissue in the absence of the BM. The underlying choroidal structure in the gamma zone PPA is distinct from its original status without gamma zone formation (Kim et al., 2018). Our data revealed that the gamma zone had a lower CC density compared to the beta zone PPA, suggesting that the CC in gamma zone would decrease first following the absence of RPE and BM. Additionally, we found that MLCV density in the gamma zone PPA was significantly lower in NPDR group compared to the NDR group. Previous studies have indicated that the peripheral choroid is more susceptible to diabetes-induced injury than the central area (Xu et al., 2023), which could explain the decrease of MLCV density adjacent to the optic disc preceding changes in the macular area. As the gamma zone PPA enlarges, the self-regulatory mechanism of MLCV compensates for the decrease in CC perfusion, maintaining the basic oxygen and nutrient requirements of the retina. This may elucidate the positive correlation observed between MLCV density and the width and area of the gamma zone PPA.

There are several limitations in the present study. Firstly, the participants enrolled in our study primarily consisted of older individuals with T2DM, therefore, our findings may not applicable to children with Type 1 Diabetes Mellitus (T1DM). Secondly, our study excluded healthy subjects without diabetes as well as subjects with proliferative diabetic retinopathy (PDR). Including these two groups in future studies could provide further information. Thirdly, all subjects were from the same center and the sample size was relatively small. Lastly, due to the inherent limitations of the cross-sectional design, we were unable to observe longitudinal changes in the microstructure and microvasculature of the beta and gamma zone PPA in diabetes with or without DR. Therefore, further exploration of the relationship between gamma zone PPA and the development of DR over time should be conducted through larger multi-center longitudinal studies.

In conclusion, our findings demonstrated that DR-induced peripapillary vascular changes primarily occur in the gamma zone PPA. After accounting for AL, our study reveals a negative correlation between DR and gamma zone PPA. Longitudinal studies are necessary to elucidate the role of the gamma zone in the development and progression of DR.

## Data Availability

The raw data supporting the conclusions of this article will be made available by the authors, without undue reservation.

## References

[B1] Alberti K. G. Zimmet P. Z. (1998). Definition, diagnosis and classification of diabetes mellitus and its complications. Part 1: diagnosis and classification of diabetes mellitus provisional report of a WHO consultation. Diabet. Med. 15 (7), 539–553. 10.1002/(SICI)1096-9136(199807)15:7<539::AID-DIA668>3.0.CO;2-S 9686693

[B2] Chhablani J. Sharma A. Goud A. Peguda H. K. Rao H. L. Begum V. U. (2015). Neurodegeneration in type 2 diabetes: evidence from spectral-domain optical coherence tomography. Investig. Ophthalmol. Vis. Sci. 56 (11), 6333–6338. 10.1167/iovs.15-17334 26436886

[B3] Dai Y. Jonas J. B. Huang H. Wang M. Sun X. (2013). Microstructure of parapapillary atrophy: beta zone and gamma zone. Investig. Ophthalmol. Vis. Sci. 54 (3), 2013–2018. 10.1167/iovs.12-11255 23462744

[B4] Guo X. Chen Y. Bulloch G. Xiong K. Chen Y. Li Y. (2023). Parapapillary choroidal microvasculature predicts diabetic retinopathy progression and diabetic macular edema development: a three-year prospective study. Am. J. Ophthalmol. 245, 164–173. 10.1016/j.ajo.2022.07.008 35863493

[B5] He K. Zhang X. Ren S. Sun J. (2016). “Deep residual learning for image recognition,” in 2016 IEEE Conference on Computer Vision and Pattern Recognition, Las Vegas, NV, June 27–30, 2016, 770–778. 10.1109/CVPR.2016.90

[B6] Hu X. Shang K. Chen X. Sun X. Dai Y. (2021). Clinical features of microvasculature in subzones of parapapillary atrophy in myopic eyes: an OCT-angiography study. Eye (Lond). 35 (2), 455–463. 10.1038/s41433-020-0872-6 32327738 PMC8027825

[B7] Isensee F. Jaeger P. F. Kohl S. A. A. Petersen J. Maier-Hein K. H. (2021). nnU-Net: a self-configuring method for deep learning-based biomedical image segmentation. Nat. Methods 18 (2), 203–211. 10.1038/s41592-020-01008-z 33288961

[B8] Jonas J. B. Jonas S. B. Jonas R. A. Holbach L. Dai Y. Sun X. (2012). Parapapillary atrophy: histological gamma zone and delta zone. PLoS One 7 (10), e47237. 10.1371/journal.pone.0047237 23094040 PMC3475708

[B9] Jonas J. B. Naumann G. O. (1989). Parapapillary chorioretinal atrophy in normal and glaucoma eyes. II. Correlations. Investig. Ophthalmol. Vis. Sci. 30 (5), 919–926. 2722448

[B10] Jonas J. B. Wang Y. X. Zhang Q. Fan Y. Y. Xu L. Wei W. B. (2016). Parapapillary gamma zone and axial elongation-associated optic disc rotation: the Beijing eye study. Investig. Ophthalmol. Vis. Sci. 57 (2), 396–402. 10.1167/iovs.15-18263 26842757

[B11] Kim M. Choung H. K. Lee K. M. Oh S. Kim S. H. (2018). Longitudinal changes of optic nerve head and peripapillary structure during childhood myopia progression on OCT: boramae myopia cohort study report 1. Ophthalmology 125 (8), 1215–1223. 10.1016/j.ophtha.2018.01.026 29550000

[B12] Kim Y. W. Lee E. J. Kim T. W. Kim M. Kim H. (2014). Microstructure of β-zone parapapillary atrophy and rate of retinal nerve fiber layer thinning in primary open-angle glaucoma. Ophthalmology 121 (7), 1341–1349. 10.1016/j.ophtha.2014.01.008 24565742

[B13] Lee E. J. Kim T. W. Weinreb R. N. Park K. H. Kim S. H. Kim D. M. (2011). β-Zone parapapillary atrophy and the rate of retinal nerve fiber layer thinning in glaucoma. Investig. Ophthalmol. Vis. Sci. 52 (7), 4422–4427. 10.1167/iovs.10-6818 21474770

[B14] Li T. Jia Y. Wang S. Xu Y. Yin Y. Wang A. (2020). Change in peripapillary and macular choroidal thickness change in children with type 1 diabetes mellitus without visual impairment or diabetic retinopathy. Acta Ophthalmol. 98 (2), e203–e211. 10.1111/aos.14225 31421015

[B15] Lin Q. Jia Y. Li T. Wang S. Xu X. Xu Y. (2022). Optic disc morphology and peripapillary atrophic changes in diabetic children and adults without diabetic retinopathy or visual impairment. Acta Ophthalmol. 100 (1), e157–e166. 10.1111/aos.14885 33949131 PMC9292269

[B16] Longo A. Geiser M. H. Riva C. E. (2004). Posture changes and subfoveal choroidal blood flow. Investig. Ophthalmol. Vis. Sci. 45 (2), 546–551. 10.1167/iovs.03-0757 14744897

[B17] Ronneberger O. Fischer P. Brox T. (2015). “U-net: convolutional networks for biomedical image segmentation,” in Medical Image Computing and Computer-Assisted Intervention—MICCAI 2015: 18th International Conference, Munich, Germany, October 5–9, 2015, proceedings, part III 18 (Berlin: Springer), 234–241. 10.1007/978-3-319-24574-4_28

[B18] Sun H. Saeedi P. Karuranga S. Pinkepank M. Ogurtsova K. Duncan B. B. (2023). IDF Diabetes Atlas: global, regional and country-level diabetes prevalence estimates for 2021 and projections for 2045. Diabetes Res. Clin. Pract. 204, 110945. 10.1016/j.diabres.2021.109119 37863776

[B19] Sun Z. Tang F. Wong R. Lok J. Szeto S. K. H. Chan J. C. K. (2019). OCT angiography metrics predict progression of diabetic retinopathy and development of diabetic macular edema: a prospective study. Ophthalmology 126 (12), 1675–1684. 10.1016/j.ophtha.2019.06.016 31358386

[B20] Sung M. S. Heo H. Park S. W. (2018). Microstructure of parapapillary atrophy is associated with parapapillary microvasculature in myopic eyes. Am. J. Ophthalmol. 192, 157–168. 10.1016/j.ajo.2018.05.022 29859144

[B21] Tan N. Y. Q. Tham Y. C. Ding Y. Yasuda M. Sabanayagam C. Saw S. M. (2018). Associations of peripapillary atrophy and fundus tessellation with diabetic retinopathy. Ophthalmol. Retina 2 (6), 574–581. 10.1016/j.oret.2017.09.019 31047611

[B22] Uchida H. Ugurlu S. Caprioli J. (1998). Increasing peripapillary atrophy is associated with progressive glaucoma. Ophthalmology 105 (8), 1541–1545. 10.1016/S0161-6420(98)98044-7 9709771

[B23] Vujosevic S. Muraca A. Gatti V. Masoero L. Brambilla M. Cannillo B. (2018). Peripapillary microvascular and neural changes in diabetes mellitus: an OCT-angiography study. Investig. Ophthalmol. Vis. Sci. 59 (12), 5074–5081. 10.1167/iovs.18-24891 30357402

[B24] Wang W. Cheng W. Yang S. Chen Y. Zhu Z. Huang W. (2023). Choriocapillaris flow deficit and the risk of referable diabetic retinopathy: a longitudinal SS-OCTA study. Br. J. Ophthalmol. 107 (9), 1319–1323. 10.1136/bjophthalmol-2021-320704 35577546

[B25] Wang Z. Hua Z. Wen Y. Zhang S. Xu X. Song H. (2024). E-YOLO: recognition of estrus cow based on improved YOLOv8n model. Expert Syst. Appl. 238, 122212. 10.1016/j.eswa.2023.122212

[B26] Wilkinson C. P. Ferris F. L. Klein R. E. Lee P. P. Agardh C. D. Davis M. (2003). Proposed international clinical diabetic retinopathy and diabetic macular edema disease severity scales. Ophthalmology 110 (9), 1677–1682. 10.1016/S0161-6420(03)00475-5 13129861

[B27] Xu F. Li Z. Yang X. Gao Y. Li Z. Li G. (2023). Assessment of choroidal structural changes in patients with pre- and early-stage clinical diabetic retinopathy using wide-field SS-OCTA. Front. Endocrinol. (Lausanne) 13, 1036625. 10.3389/fendo.2022.1036625 36743939 PMC9892628

[B28] Yau J. W. Rogers S. L. Kawasaki R. Lamoureux E. L. Kowalski J. W. Bek T. (2012). Global prevalence and major risk factors of diabetic retinopathy. Diabetes Care 35 (3), 556–564. 10.2337/dc11-1909 22301125 PMC3322721

[B29] You Q. S. Wang J. Guo Y. Pi S. Flaxel C. J. Bailey S. T. (2020). Optical coherence tomography angiography avascular area association with 1-year treatment requirement and disease progression in diabetic retinopathy. Am. J. Ophthalmol. 217, 268–277. 10.1016/j.ajo.2020.04.024 32360332 PMC7492451

[B30] Zhang Q. Wang Y. X. Wei W. B. Xu L. Jonas J. B. (2018). Parapapillary beta zone and gamma zone in a healthy population: the Beijing eye study 2011. Investig. Ophthalmol. Vis. Sci. 59 (8), 3320–3329. 10.1167/iovs.18-24141 30025091

